# Body-related concerns and participation in physical education among adolescent students: the mediating role of motivation

**DOI:** 10.3389/fpsyg.2023.1266740

**Published:** 2023-09-22

**Authors:** Ellen Haug, Isabel Castillo, Oddrun Samdal, Otto Robert Frans Smith

**Affiliations:** ^1^Department of Health Promotion and Development, University of Bergen, Bergen, Norway; ^2^Department of Teacher Education, NLA University College, Bergen, Norway; ^3^Department of Social Psychology, University of Valencia, Valencia, Spain; ^4^Department of Health Promotion, Norwegian Institute of Public Health, Bergen, Norway

**Keywords:** body-related concerns, motivation, physical education, physical activity, adolescents

## Abstract

**Background:**

There is a need to understand better factors influencing participation in physical education (PE) and the mechanisms involved. The adolescent years are characterised by increasing levels of body-related concerns. In PE, the body is judged for its physical abilities and subject to social comparisons and body judgements. Grounded in the Self-Determination Theory, this study aimed to explore whether body-related factors were associated with adolescents’ involvement in PE and whether types of motivation mediated this relationship.

**Methods:**

The study involved 2,140 (54.5% girls) secondary students (15–16-year-olds) from Norway participating in the nationally representative “Health Behaviour in School-aged Children (HBSC) study: a WHO collaborative cross-national study.” Body-related factors included Body Mass Index (BMI), health complaints, body perception and dietary behaviours. Gender, age, and socioeconomic status (family affluence) were control variables. Motivation for PE was assessed with the Perceived Locus of Causality (PLOCQ) scale measuring three distinct factors: autonomous motivation, controlled motivation and amotivation. PE involvement was self-reported as weekly participation in PE classes and time spent in moderate-to-vigorous physical activity (MVPA) during PE.

**Results:**

Gender (girl), family affluence, health complaints, not being on a diet but wanting to lose weight, and body perception (too fat) were negatively associated with weekly PE participation when adjusting for other variables. This association was largely explained by students’ autonomous motivation in the case of health complaints and partly in the case of dietary behaviour and body perception. Similar results were observed for MVPA during PE lessons. Additionally, gender was associated with MVPA through amotivation.

**Conclusion:**

The study adds new knowledge to the understanding of the relationship between body-related factors and PE, supporting that autonomous motivation is a central mechanism and an avenue for further research. The results should be considered in planning high-quality PE classes and suggest that an autonomous supportive learning climate sensitive to body-related concerns should be a priority to increase adolescent involvement in PE.

## Introduction

A physically active lifestyle is associated with numerous physical and mental health benefits for adolescents (Chaput et al., 2020). It is recommended for this age group to do at least an average of 60 min per day of moderate-to-vigorous physical activity (MVPA), primarily aerobic, across the week (World Health Organization, 2020). However, existing data suggest that a minority of adolescents are sufficiently physically active, and the activity level decreases with increasing age (Inchley et al., 2020; World Health Organization, 2022). The school setting, and in particular the physical education (PE) context, has been given an essential role in providing enjoyable, educational physical activity that can foster the development of motor skills and introduce students to a variety of activities that can be performed in a life-time perspective (World Health Organization, 2018a,b). The PE setting has the potential to offer a structured environment and learning activities that can influence students’ physical activity (PA) behaviours in PE lesson time and during leisure time (Owen et al., 2014; Castillo et al., 2020; Vasconcellos et al., 2020). It has also been documented that positive attitudes towards PE can upheld over time (Ladwig et al., 2018) and predict future PA participation (Rhodes and Kates, 2015).

Nevertheless, available data suggest that students’ time spent in MVPA during PE classes varies substantially, with a decrease observed from middle school to high school (Hollis et al., 2017). The age-related decrease co-occurs with an overall decline in motivation for PE (Papaioannou et al., 2006; Yli-Piipari et al., 2009). Motivational processes in PE are multifaceted and complex, with motivational influences divided into internal and external aspects (Kretschmann, 2014). Internal factors comprise individual characteristics (e.g., gender, physical attributes), dispositional variables (e.g., perceived competence and autonomy), and individual situational variables (e.g., sports involvement). External factors include environmental, situational variables (e.g., teachers’ skills and teaching style) and contextual variables (e.g., PE curriculum, PE activities) (Kretschmann, 2014). Thus, to develop quality PE programmes, there is a need to better understand factors at multiple levels associated with students’ engagement and participation.

So far, research efforts on motivation and participation in PE have primarily focused on addressing the subject from a social-psychological perspective, with the Self-Determination Theory (SDT) (Deci and Ryan, 1985, 2000) as the most used theoretical framework (Lindahl et al., 2015). In this regard, much of the existing research has investigated the role of autonomy-supportive environments on motivational processes, focusing on teachers’ role in creating a motivational climate (Vasconcellos et al., 2020). However, in PE classes, the body is at the centre of curricular outcomes. It is being judged for physical abilities and positioned in ways that open for social comparisons and body judgements (Kerner et al., 2018). Thus, the current study aims to extend the existing research by examining the link between individual characteristics and PE participation, focusing on body-related concerns among adolescents. Grounded in the SDT, special attention is given to the mediation pathway of motivation forms.

SDT distinguishes between different types of motivations varying on a continuum from controlled to more autonomous forms, with an increasing degree of self-determination present (Ryan and Deci, 2017). According to SDT, *intrinsic motivation* is described as the inherent propensity to develop skills actively, engage in challenges, and take an interest in new activities (Deci and Ryan, 1985). Extrinsic motivation is divided into four types of regulation: integrated, identified, introjected and external (Ryan and Deci, 2017). *Integrated regulation* represents the most self-determined form and refers to behaviours executed out of choice to harmonise and bring coherence to different parts of the self. *Identified regulation* refers to a situation when the behaviour is highly valued by the individual and, therefore performed with less pressure, even if it is not particularly pleasant. *Introjected regulation* refers to behaviour starting to be internalised but not entirely self-determined. This kind of behaviour may be performed to gain social recognition or avoid feelings of guilt. *External regulation* is behaviour regulated through external means, such as punishment or rewards. The last category is amotivation which refers to the absence of motivation.

SDT proposes that intrinsic motivation and the more autonomous types of extrinsic motivation will lead to constructive functioning, better learning and improved psychological health and well-being (Ryan and Deci, 2017). Literature from the PE context has demonstrated that self-determined forms of motivation towards PE are positively associated with several desirable responses in physical education, such as greater concentration, effort, and persistence (Ntoumanis, 2001; Standage et al., 2005), higher levels of positive affect (Ntoumanis, 2005; Standage et al., 2005; Zamarripa et al., 2016), physical activity in PE (Owen et al., 2014), participation in PE (Owen et al., 2014; Ulstad et al., 2019), and higher levels of leisure time PA as well as greater intention to continue to be physically active (Castillo et al., 2020). Also, introjected regulation has been weakly associated with PA in PE lessons (Owen et al., 2014). Amotivation has, on the other hand, been positively associated with unhappiness, negative affect, and boredom in PE (Ntoumanis, 2001; Standage et al., 2005; Zamarripa et al., 2016; White et al., 2021).

Physical perceptions and experiences in PE can be impediments to students’ motivation towards PE (Papacharisis and Goudas, 2003), and several qualitative studies have identified body-related issues as obstacles to participating in PE and sports (Allender et al., 2006; Martins et al., 2021). Many studies suggest that embarrassment, body image concerns, physical discomfort and body insecurity are barriers to PE and PA participation and are more frequently reported among girls than boys (Martins et al., 2021; White et al., 2021). Body image is a multidimensional construct that includes how one sees, thinks, feels, and behaves related to the body’s appearance and function (Cash and Smolak, 2011). An enhanced body image has been positively associated with engagement in PE (Bevans et al., 2010) and with physical activity (Sabiston et al., 2019; Martins et al., 2021). The adolescent years are characterised by body changes taking place and the internalisation of the society-imposed aesthetic model (Paxton et al., 2005). The cognitive dimension of body image, body dissatisfaction, is reflected in the desire of someone to lose weight or to gain weight (Laus et al., 2011). Body dissatisfaction reaches high levels in this period, especially among females (Fernández-Bustos et al., 2019), with clear gender differences observed (Laus et al., 2011; Sanchez-Miguel et al., 2021). In a time of high prevalence of body image disturbances among adolescents (Dion et al., 2016), it is pertinent to understand better how body-related factors are associated with physical activity in the PE context.

In addition to gender, weight status, BMI, and physical ability have been identified as key variables for body dissatisfaction (Dion et al., 2016; Fernández-Bustos et al., 2019; Molina-García et al., 2019). Evidence suggests that many overweight students report negative experiences of PE, such as bullying and embarrassment, along with other barriers to participation (Fox and Edmunds, 2000; Cardinal et al., 2014). As a result, this may lead to maladaptive coping behaviours, such as avoiding PA and PE (Li et al., 2017). However, improving body shape, physical appearance and weight management have also been reported as reasons for participation in physical activities, especially among girls’, suggesting that more external forms of regulations may drive involvement in PE (Martins et al., 2021). For example, some qualitative studies have reported that pressure to conform to popular beauty ideals is important reason for teenage girls to be physically active (Allender et al., 2006). Restrictive dieting and weight control are also frequently used by adolescents attempting to achieve an internalised image of an ideal body (Krowchuk et al., 1998).

Other body-related aspects that can affect the individual student and may be related to participation in PE are subjective health complaints. Such complaints can have both a somatic (e.g., headache and backache) and a psychological (e.g., feeling low and feeling nervous) dimension that is not explained by an underlying illness (Brown, 2007). Subjective health complaints have become progressively prevalent in children, increase with age (Inchley et al., 2020) and have been related to absenteeism in school (Saps et al., 2009) and lower PA levels (Marques et al., 2015; Keane et al., 2017). The impact of health complaints in the PE context has to our knowledge, not been examined. As the PE lessons might demand a well-functioning body focusing on performing in front of others, competition, grading and unrealistic standards (White et al., 2021), such complaints may be an impediment to experiencing autonomous motivation and thus impact involvement in PE.

SDT and motivational processes have been suggested as a potential avenue for further research to explain the relationship between body-related factors and PA (Vani et al., 2021). Based on a relatively large Norwegian nationally representative sample of secondary school students, the current study aimed to test a model of the relationship between body-related factors (i.e., BMI, health complaints, body perception, weight control behaviours) and measures of PE involvement, examining the mediating role of motivation in this relationship.

## Materials and methods

### Participants and procedure

The data stem from a national sample (*n* = 2,140) of Norwegian lower and upper secondary school students aged 15 and 16 years (59%) participating in the 2013/2014 survey of the “Health Behaviour in School-aged Children (HBSC) study: a WHO collaborative cross-national study.” The participants had a mean age of 16.3 ± 0.7 years, and 51% was a girl. This correlational study with a non-experimental, quantitative, and cross-sectional design had school class as the primary sampling unit. The classes were chosen from a geographically stratified list to ensure a nationally representative sample. The Norwegian Western Regional Ethical Committee (REK) approved the study and the use of passive consent (2013/1494/REK vest). A detailed information letter was given both in paper form and electronically to parents or custodians. Those who did not want their child to participate had to sign and return a form to the teacher. Approval of the child’s participation was assumed if the form was not returned. The class teachers administered the survey between January 2014 and May 2014. Participation was voluntary, and the anonymity, as well as the confidentiality of the participants, were ensured. The participants could withdraw from the study at any time.

### Measures

#### Exposures

BMI was calculated (in kilogram per square meter) based on self-reported weight and height measured by the questions: “How much do you weigh without clothes?” and “How tall are you without shoes?” Self-reported height and weight are considered suitable measures for detecting valid relationships in epidemiological studies (Spencer et al., 2002; Aasvee et al., 2015).

Health complaints were assessed with the HBSC Symptom Checklist (HBSC-SHC) (Haugland and Wold, 2001; Ravens-Sieberer et al., 2008). The participants were asked how often they had experienced the following during the past 6 months; headache, stomach-ache, feeling dizzy, feeling low, irritability or bad temper, feeling nervous, and difficulties in getting to sleep. The first four health complaints are defined as somatic and the latter four as psychological health complaints. The response options were “about every day,” “more than once a week,” “about every week,” “about every month,” and “rarely or never any symptoms.” For the present study, health complaints were modelled as a mean sum score. The HBSC-SHC has adequate test–retest reliability and validity properties (Haugland and Wold, 2001).

Body perception, an appearance facet of body image (Vani et al., 2021), was assessed with the question “Do you think your body is: “much too thin,” “bit too thin,” “about the right size,” “bit too fat” or “much too fat.” The latter two response options were recoded as “too fat” and the first two responses where coded as “too thin.” The test–retest stability in self-perceived weight has been found to be excellent (ICC = 0.81; 95%  CI = 0.76–0.85) (Currie et al., 2001).

To identify weight control behaviours, participants were asked to indicate if they were at present on a diet or doing something to lose weight. Possible responses were “Yes”; “No, but I should lose some weight”; “No, my weight is fine”; and “No, because I need to put on weight”.

As control variables, family affluence, a dimension of socioeconomic status, was assessed using the family affluence scale (FAS-III) (Hartley et al., 2016). FAS is a measure of material affluence derived from the characteristics of the family’s household and consists of six items (family car, number of computers, own bedroom, family holidays, number of bathrooms, dishwasher in home). A sum score was calculated to range from 0 (low material affluence) to 13 (high material affluence). Gender was measured as either boy or girl. The participant also reported the month and year of birth, which was then calculated based on the survey completion time. After rounding to the nearest age group, they were subsequently grouped as 15- and 16-year-olds.

### Mediators

The Perceived Locus of Causality (PLOCQ) (Aasvee et al., 2015) was employed to examine students’ motivational regulations towards PE at a contextual level. Each motivational regulation comprised four items following the heading “Why do you participate in Physical Education?” and the stem “I take part in PE classes.” The subscales in the questionnaire intended to measure intrinsic motivation (e.g., “because PE is fun”), identified regulation (e.g., “because it is important for me to do well in PE”), introjected regulation (e.g., ‘because I would feel bad about myself if I did not), external regulation (e.g., ‘because I’ll get into trouble if I do not), and amotivation (e.g., ‘but I do not see why we should have PE’). Responses were reported on a seven-point scale ranging from 1 (strongly disagree) to 7 (strongly agree). The scale was translated into Norwegian, and then back-translated to English following the procedures from the HBSC-study protocol (Currie et al., 2014). The reliability and validity of the subscale scores of the PLOCQ have generally been supported. However, there have been concerns regarding internal consistency and discriminant validity for some of the subscales. More specifically, the self-determined motives (i.e., intrinsic motivation and identified regulation) have not been distinguishable by youth across several studies and cultures (Ntoumanis, 2005; Lonsdale et al., 2011). In line with previous studies (Koestner et al., 2008; Alvarez et al., 2021), we adopted a 3-factor model in which the items reflecting intrinsic and identified regulation were combined into a sum scored autonomous motivation factor, and the items reflecting introjected and external regulation were combined into a sum scored controlled motivation factor. The third factor we used was sum scored amotivation. For estimation purposes the sum scores were transformed to mean scores ranging from 1 to 7.

### Outcomes

Frequency of weekly physical education classes was assessed with the question, “How many times in a regular week do you participate in physical education classes? (also include elective classes, e.g., sports and outdoor life”). This was followed by the specification, “A one 90-min class should count as two times.” The response categories were (1) 0–1 time, (2) 2 times, and (3) more than two times.

Duration of MVPA in physical education was a measure of physical activity of moderate to vigorous intensity derived from the HBSC-item on overall MVPA (Prochaska et al., 2001). The wording was “How many minutes in a single PE class (45 min) do you usually perform physical activity in a way that makes you warm and out of breath?.” The response categories were labelled from (1) 0 min, (2) 1–10 min, (3) 11–20 min, (4) 21–30 min, and (5) more than 30 min. In the analysis, these were reduced to three categories: (1) less than or equal to 20 min, (2) 21–30 min, and (3) more than 30 min.

### Ethical considerations

The study was conducted according to the guidelines of the Declaration of Helsinki and the Norwegian Western Regional Ethics Committee (REK) approved the study and the use of passive consent. A detailed information letter was given in paper form and electronically to parents or custodians. Those who did not want their child to participate had to sign and return a form to the teacher. Approval of the child’s participation was assumed if the form was not returned. Participation was voluntary, and the anonymity, as well as the confidentiality of the participants, were ensured. The participants could withdraw from the study at any time.

### Statistical analyses

**Figure 1:**
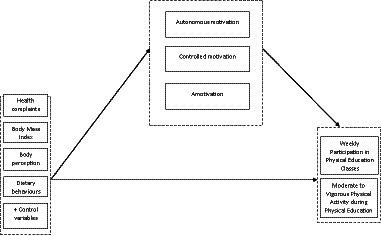
Conceptual diagram of the proposed parallel multiple mediator model.

The mediation model (see Figure 1) was estimated by a series of regression equations. The three mediators were regressed on the exposure variables using linear regression, whereas the outcome variables were regressed on the mediators and the exposure variables using ordinal regression. The outcome variables were also regressed on the exposure variables without the inclusion of the mediator variables to examine more directly the total effects of the exposure variables on PE participation and MVPA. Indirect effects were calculated by Mplus as the product of the exposure-mediator regression coefficient and the mediator-outcome regression coefficient. As the outcomes were ordinal, Mplus uses their underlying latent response variables to calculate the (in)direct effects. Robust full information maximum likelihood with Monte Carlo integration was used as estimator in Mplus version 8.8.

## Results

### Descriptive statistics

Descriptive statistics for the study’s variables are presented in Table 1. The participants exhibited the highest average scores of autonomous motivation and the lowest average scores of amotivation. The participants reported most frequently participating in PE classes twice a week and MVPA levels during PE of more than 30 min.

**Table 1:** Descriptive statistics.

Gender, % (*n*)	
Boys	45.5 (973)
Girls	54.5 (1167)
Age group, % (*n*)	
15-year-olds	41.0 (878)
16-year-olds	59.0 (1262)
Family affluence scale, mean (SD)	10.0 (1.6)
Health complaints, mean (SD)	2.1 (8.4)
Body Mass Index, mean (SD)	21.4 (3.3)
Dieting variable, % (*n*)	
On a diet	25.8 (521)
Not on a diet, need to lose weight	14.4 (291)
Not on a diet, need to put on weight	12.0 (241)
Not on a diet, weights fine	47.8 (963)
Body perception, % (*n*)	
Too fat	41.0 (821)
Too thin	13.3 (268)
About right	45.6 (912)
Motivation form, mean (SD)	
Autonomous motivation	4.9 (1.8)
Controlled motivation	4.4 (1.4)
Amotivation	2.5 (1.6)
Weekly participation in PE Classes, % (*n*)	
0–1 time	17.4 (336)
Twice	53.0 (1023)
More than twice	29.6 (572)
MVPA during PE, % (*n*)	
≤ 20 min	25.8 (493)
21–30 min	26.6 (508)
> 30 min	47.6 (911)

### Mediation model for body-related factors, motivation, and participation in physical education

In the model in which the outcome variables were regressed on the exposure variables without the inclusion of the mediator variables, the following variables were significantly associated with PE participation: Age, family affluence, being on a diet, not being on a diet but wanting to lose weight and health complaints. The exposures age, family affluence, and health complaints were associated with MVPA (see Table 2).

**Table 2:** Unstandardized regression coefficients of the proposed mediation model (adjusted estimates).

Exposures	Mediators			Outcomes			
	Autonomous motivation *b* (se)	Controlled motivation *b* (se)	Amotivation *b* (se)	PE participation *b* (se)		MVPA during PE *b* (se)	
	–	–	–	Not controlled for motivation	Controlled for motivation	Not controlled for motivation	Controlled for motivation
Gender: girl	−0.23 (0.09)*	0.05 (0.07)	−0.39 (0.10)***	−0.19 (0.12)	−0.15 (0.12)	−0.13 (0.10)	−0.10 (0.10)
16 -year-olds	−0.07 (0.11)	0.19 (0.08)*	0.06 (0.10)	−0.91 (0.18)***	−0.93 (0.18)***	0.27 (0.12)*	0.31 (0.11)**
Family affluence	0.10 (0.03)**	0.07 (0.02)**	0.03 (0.03)	0.09 (0.03)*	0.06 (0.03)	0.08 (0.03)**	0.05 (0.03)
Health complaints	−0.51 (0.06)***	−0.11 (0.05)*	0.19 (0.05)***	0.24 (0.07)***	−0.07 (0.06)	0.24 (0.06)***	−0.07 (0.07)
BMI	−0.03 (0.02)	−0.03 (0.01)*	−0.00 (0.01)	−0.00 (0.02)	0.01 (0.02)	0.00 (0.02)	. 02 (0.02)
On a diet	−0.21 (0.11)	0.28 (0.10)**	0.17 (0.10)	−0.29 (0.12)*	−0.26 (0.12)*	−0.14 (0.13)	−0.07 (0.15)
Not on a diet, need to lose	−0.60 (0.13)***	−0.16 (0.09)**	0.21 (0.12)	−0.50 (0.14)***	−0.34 (0.14)*	−0.34 (0.15)	−0.15 (0.15)
Not on a diet, need to put on	−0.30 (0.16)	0.01 (0.15)	0.20 (0.16)	−0.00 (0.20)	0.07 (0.29)	0.02 (0.18)	0.13 (0.19)
Body perception, too thin	−0.11 (0.16)	−0.04 (0.15)	−0.13 (0.15)	−0.33 (0.18)	−0.34 (0.18)	−0.09 (0.17)	−0.07 (0.18)
Body perception, too fat	−0.61 (0.17)***	−0.40 (0.17)*	0.27 (0.16)	−0.15 (0.20)	0.05 (0.20)	−0.30 (0.18)	−0.13 (0.19)
Autonomous motivation					0.31 (0.05)***		0.31 (0.03)***
Controlled motivation					0.04 (0.05)		0.07 (0.03)*
Amotivation					−0.03 (0.04)		−0.07 (0.03)*
R-square	0.14	0.04	0.03	0.10	0.17	0.04	0.12

PE, physical education; MVPA, moderate-to-vigorous physical activity. BMI, body mass index. Reference categories: gender; boy, age; 15-year-olds, dietary behaviour; Not on a diet the weight is just fine, body perception; about the right size. ^***^ *p* < 0.001, ^**^ *p* < 0.01, ^*^ *p* < 0.05.

After including the mediator variables in the outcome model, most of the associations mentioned above became smaller, with the expectation of the association with age. As shown in Table 2, students with higher levels of autonomous motivation reported participating in PE classes more often and with higher levels of MVPA when controlling for gender, age, family affluence, body-related factors and other specific forms of motivation. Similarly, controlled motivation was positively associated with MVPA during PE, whereas students with higher levels of amotivation reported lower levels of MVPA. However, these associations were weaker than for autonomous motivation. Table 2 shows that being a girl, having lower family affluence, having more health complaints, not being on a diet but wanting to lose weight, and perceiving the body as too fat were associated with lower levels of autonomous motivation. On the other hand, 16-year-olds reported higher levels of controlled motivation than 15-year-olds. Being on a diet and having higher family affluence was also associated with higher levels of controlled motivation. A higher BMI, more health complaints, not being on a diet but wanting to lose weight, and perceiving the body as too fat were associated with lower levels of controlled motivation. Finally, females reported lower levels of amotivation, whereas students with higher levels of health complaints reported higher levels of amotivation.

Examination of indirect effects suggested that gender, family affluence, dietary behaviour, perceiving the body as too fat and health complaints were associated with PE participation through autonomous regulation motivation (see also Table 3). For perceiving the body as too fat and health complaints, the indirect effect explained most of the total effect of these variables on PE participation. The results were largely similar for MVPA, but additionally, gender was associated with MVPA through amotivation. Age group was the only exposure variable with a statistically significant total effect that was not explained at all by any form of motivation.

**Table 3:** Unstandardized indirect, direct, and total effects†.

Exposure	Mediator	Outcome	Indirect effect (95% CI)	Direct effect (95% CI)	Total effect (95% CI)
Gender girl	Autonomous	Weekly PE participation	−0.07 (−0.13, −0.01)*	−0.15 (−0.38, 0.08)	−0.21 (−0.45, 0.03)
Family affluence	Autonomous	Weekly PE participation	0.03 (0.01, 0.05)**	0.06 (−0.01, 0.12)	0.09 (0.02, 0.16)*
Health complaints	Autonomous	Weekly PE participation	−0.16 (−0.23, −0.09)***	−0.07 (−0.20, 0.05)	−0.24 (−0.38, −0.11)***
Not on a diet, need to lose	Autonomous	Weekly PE participation	−0.19 (−0.29, −0.09)*	−0.34 (−0.62, −0.07)*	−0.55 (−0.84, −0.25)***
Body perception, too fat	Autonomous	Weekly PE participation	−0.19 (−0.32, −0.06)**	0.05 (−0.33, 0.43)	−0.16 (−0.58, 0.25)
Gender girl	Autonomous	MVPA during PE	−07 (−0.13, −0.01)*	−0.10 (−0.30, 0.11)	−0.14 (−0.34, 0.07)
Gender girl	Amotivation	MVPA during PE	0.03 (0.00, 0.05)*	−0.10 (−0.30, 0.11)	−0.14 (−0.34, 0.07)
Family affluence	Autonomous	MVPA during PE	0.03 (0.01, 0.05)**	0.05 (−0.01, 0.11)	0.08 (0.02, 0.15)*
Health complaints	Autonomous	MVPA during PE	−0.16 (−0.21, −0.10) ***	−0.07 (−0.19, 0.06)	−0.24 (−0.38, −0.11)***
Not on a diet, need to lose	Autonomous	MVPA during PE	−0.19 (−0.27, −0.10)***	−0.15 (−0.45, 0.16)	−0.36 (−0.67, −0.04)*
Body perception, too fat	Autonomous	MVPA during PE	−0.19 (−0.30, −0.07)**	−0.13 (−0.51, 0.25)	−0.36 (−0.75, 0.03)

^***^ *p* < 0.001, ^**^ *p* < 0.01, ^*^ *p* < 0.05, †only pathways with statistically significant indirect effects are displayed.

## Discussion

This study aimed to increase our understanding of the link between body-related concerns and involvement in PE, with forms of motivation as the mediating pathway. In line with the tenets of SDT (Ryan and Deci, 2017), students with autonomous motivation attended PE classes more frequently and reported higher levels of MVPA during PE lessons when other factors were adjusted for. Controlled motivation was positively and amotivation negatively associated with MVPA during PE. The results indicated that the associations between body-related concerns and PE participation/MVPA were partly explained by autonomous motivation. More specifically, we found that the association between health complaints and PE participation/MVPA was almost fully explained by autonomous motivation, whereas the association with dietary behaviour (not on a diet but wanting to lose weight) and body perception (too fat) were partly explained by autonomous motivation.

The study finding of positive associations between autonomous motivation with both weekly participation in PE and MVPA during PE lessons adds to studies that have demonstrated adaptive outcomes of autonomous motivation in the PE context (Ntoumanis, 2005; Standage et al., 2005; Owen et al., 2014; Zamarripa et al., 2016; Lonsdale et al., 2019; Vasconcellos et al., 2020). For weekly participation in PE, the students were asked to report mandatory PE and self-selected optional PE courses. This might partly reflect a self-selection for optional PE courses among those reporting higher levels of autonomous motivation. This would align with the prospective study by Ntoumanis (2005), which found that students who chose to enrol in optional PE courses reported higher levels of self-determined motivation and lower levels of amotivation than those who decided not to enrol.

Controlled motivation was, in addition, a positive predictor and amotivation a negative predictor of MVPA during PE lessons, suggesting partly differential motivational processes for attending PE classes and MVPA during PE. In Owen et al. (2014) meta-analysis, a weak positive effect was also found between introjected regulation and PA in PE lessons. According to the SDT, the more controlled regulations (i.e., introjected and external regulations) are either driven by external demands to avoid negative reactions or for rewarding reasons (Ryan and Deci, 2017). A positive link between controlled motivation and MVPA during PE lessons could therefore be explained by the fact that students are being graded based on their accomplishments and efforts.

Ratelle et al. (2007) propose that the extrinsic controls and rigid constraints entailed in school could explain why students developed controlled forms of motivation. In line with this reasoning, these authors identified a student profile with high autonomous and controlled motivation as the most favourable for outcomes such as high persistence and achievement, low absenteeism, and high cognitive and affective functioning. Nevertheless, the effects on PA of more controlled regulations in PE are not likely to be upheld over time (Haerens et al., 2010). A weak negative association between amotivation and MVPA in PE is also in accordance with the meta-analysis of Owen et al. (2014) and the tenets of SDT (Ryan, 2017). Amotivation has been positively associated with unhappiness, negative affect, and boredom in PE (Ntoumanis, 2001; Standage et al., 2005; White et al., 2021), which may directly impact students’ efforts and intensity levels during PE, and, therefore, their MVPA levels.

Health complaints, not being on a diet but wanting to lose weight, and body perception (too fat) were negatively associated with weekly participation in PE when adjusting for other variables. This association was largely explained by students’ autonomous motivation in the case of health complaints and partly in the case of dietary behaviour and body perception. Similar results were observed for MVPA during PE lessons. The mechanisms involved in the observed relationships can be understood in light of a central proposition within the SDT, postulating that self-determined forms of motivation depend on the fulfilment of three innate basic psychological needs; the needs for competence, relatedness and autonomy (Ryan and Deci, 2017). Empirical work within the PE context has shown that the three needs predict autonomous motivation independently and combined (Standage et al., 2007). Within the SDT, Cognitive Evaluation Theory (Deci and Ryan, 1985) states that social factors perceived as controlling for the individual are likely to influence the basic psychological needs and, consequently, levels of self-determined motivation. Although not thoroughly studied, it has been suggested that different dimensions of body-related concerns may impact motivation in PE by undermining the psychological need satisfaction (Gillison et al., 2011). For instance, previous studies have found perceived competence satisfaction to be the strongest predictor of autonomous forms of motivation in PE compared to perceived autonomy and relatedness (Standage et al., 2005; Taylor et al., 2010). It is likely that with increased somatic or psychological health complaints, students might feel unable to meet PE demands, which can cause other adverse affective outcomes (White et al., 2021). It is also likely that the relationship between health complaints and physical activity is bidirectional (Marques et al., 2015). Nevertheless, experiencing somatic and psychological symptoms may impact the evaluation of the body’s physical features and the capacity to perform in PE. Overall, the findings add to previous research that has demonstrated an inverse relationship between health complaints and physical activity among adolescents (Marques et al., 2015; Keane et al., 2017).

Also, perceived external pressure on body image could compromise the need for autonomy, whereas the concern that others are judging one’s body shape negatively compromises the need for relatedness (Pelletier et al., 2001). Avoidance has been identified as one key coping strategy for managing negative experiences of the body (Li et al., 2017). It has been suggested that the PE non-participants, or those students who regularly disengage with PE, undertake such avoidance strategies to reduce the risk of body image disruption (Kerner et al., 2018). Past studies have also demonstrated that students with high BMI report lower perceptions of physical competence and social relatedness than those with lower BMI (Carissimi et al., 2017; Gråstén and Watt, 2017). Interestingly, BMI was not a unique predictor of motivation forms or PE in the current study that also included dietary behaviours and body perception.

Ideally, the PE context could serve as an arena to increase body appreciation (Vani et al., 2021). An enhanced body image has been positively associated with engagement (Bevans et al., 2010) and PA (Sabiston et al., 2019; Martins et al., 2021) in PE. However, it has been argued that aspects related to different dimensions of body image in PE may appear to be like the ‘elephant in the room’: obvious yet overlooked or ignored, not only in research but also in practice (Kerner et al., 2018). Nevertheless, if the learning climate in PE supports rather than thwarting basic psychological needs through teaching style and activities (White et al., 2021), it could make a difference for students with various body-related concerns. A recent review of qualitative studies has elucidated our understanding of how the satisfaction and frustration of different needs in the PE context can affect multiple outcomes, such as participation in PE (White et al., 2021). A qualitative study of Norwegian 15-year-old girls supports the association between body perceptions and PE participation (Walseth et al., 2017). Therefore, more research is needed to advance conceptual and theoretical understanding of the complex relationship between body-related factors, gender, age, motivation forms and participation in PE.

The examination of indirect effects suggested that also gender and family affluence were associated with PE participation through autonomous motivation. The results were largely similar for MVPA during PE, but additionally, gender was associated with MVPA through amotivation. PE in Norway is a coeducational setting, with both girls and boys attending the same class. In coeducational settings, girls have reported feeling that their bodies are under inspection by boys, leading to increases in body anxiety (Flintoff and Scraton, 2001). Studies have also found that girls place a higher value on their physical appearance in the coeducational context (O'Donovan and Kirk, 2008). Similar findings of girls’ body discomfort in PE classes were identified by Walseth et al. (2017) in the Norwegian qualitative study of 15-year-old girls. In the review of White et al. (2021), several factors were listed as contributes to amotivation. These were boredom from repeatedly doing the same activities, teacher-created performance climate, dominance of peers, and grades, all of which can thwart the need for competence, relatedness and autonomy. Overall, the findings suggest that teacher-created learning climates that nurture autonomous motivation and are sensitive to gender preferences seem relevant. Interestingly, the age group was the only exposure variable with a statistically significant total effect that was not explained at all by any of the motivation variables. The negative association with weekly PE participation can be explained by fewer mandatory PE classes from lower to upper secondary school. Also, upper secondary is not compulsory schooling in Norway. It could also be that 16-year-olds may have stronger interest in the health outcomes of being physically active. However, the positive association between age and MVPA during PE classes was unexpected and should be further explored but could relate to curriculum content.

### Strengths and limitations

A strength of the current study is the use of a representative national sample of adolescent students and established and validated measures on body-related factors, including health complaints, family affluence and motivation in PE. However, some limitations should be acknowledged. First, the measure included to assess MVPA levels during PE was self-reported, known to have recall and reporting bias (Nigg et al., 2020). Almost half of the students reported MVPA “more than 30 min”, which may suggest an overestimation, as other studies using more objective methods of measuring PA have found that the level of MVPA in a 45- minutes PE lesson is usually less than 50% of the lesson time duration (Owen et al., 2014). Thus, future studies should include objective measures of physical activity (e.g., accelerometers) to help confirm the current findings. Also, research suggests that MVPA in PE lessons depends on the type of activities during PE and the intensity profiles of the PE lessons (Zhou and Wang, 2019). This information was not available in the current study. Such differentiation could have given more detailed knowledge of the impact of motivation on the relationship between body-related aspects and adolescent PE behaviour across PE lesson contexts. Further, given the cross-sectional design, although the hypothesised relationship direction aligns with motivation theory, causal inferences cannot be drawn. Finally, additional research directions within the framework of SDT could have offered a more fine-grained understanding of how body-related aspects are associated with adolescent PE behaviour, for example, by including the sequence of BPB in the model. However, measures of BPN in PE were not included in this survey.

## Conclusion

This study extends the research on factors influencing adolescents’ engagement in PE by demonstrating an indirect relationship between gender, dietary behaviours, body perception, health complaints and PE, with, to a large extent, autonomous motivation as the mediating pathway. The study supports using SDT as a promising avenue for further research on body-related factors and PA participation in the PE context. The findings suggest that need supportive and a motivational learning climate sensitive to body-related aspects should be a priority when planning high-quality PE classes, especially during the adolescent years, a critical period for bodily concerns. As students with lower levels of autonomous motivation participate less often in PE, this group can potentially miss out on the educational, developmental, and health-related outcomes that school-based PE may provide.

## Data availability statement

The datasets presented in this article are not readily available. The University of Bergen is the data-bank manager for the international HBSC study. The data from the 2013/2014 survey is open access and available upon request. Requests to access the datasets should be directed to https://www.uib.no/en/hbscdata/113290/open-access.

## Ethics statement

This study involving humans were approved by The Norwegian Western Regional Ethics Committee (REK). The study was conducted in accordance with the local legislation and institutional requirements. Written informed consent for participation was not required from the participants or the participants’ legal guardians/next of kin in accordance with the national legislation and institutional requirements.

## Author contributions

EH: Conceptualization, Investigation, Methodology, Writing – original draft. IC: Writing – review & editing. OS: Data curation, Writing – review & editing. ORFS: Formal analysis, Writing – review & editing, Conceptualization, Investigation.
